# 544 Unlocking Midazolam’s Potential as a Biomarker for CYP3A4 Phenoconversion in Pediatric Burn and Surgery Patients

**DOI:** 10.1093/jbcr/irae036.178

**Published:** 2024-04-17

**Authors:** Vincent Basas, Kristin N Grimsrud, Emma Sherman, Tina L Palmieri

**Affiliations:** Shriners Children's Hospital, Sacramento, CA; University of California, Davis, Davis, CA; University of California, Davis, Sacramento, CA; UC Davis, Shriners Children's Northern California, Sacramento, CA; Shriners Children's Hospital, Sacramento, CA; University of California, Davis, Davis, CA; University of California, Davis, Sacramento, CA; UC Davis, Shriners Children's Northern California, Sacramento, CA; Shriners Children's Hospital, Sacramento, CA; University of California, Davis, Davis, CA; University of California, Davis, Sacramento, CA; UC Davis, Shriners Children's Northern California, Sacramento, CA; Shriners Children's Hospital, Sacramento, CA; University of California, Davis, Davis, CA; University of California, Davis, Sacramento, CA; UC Davis, Shriners Children's Northern California, Sacramento, CA

## Abstract

**Introduction:**

Burn patients, due to hypermetabolism, organ failure, protein binding alterations, inflammatory responses, drug interactions, and other comorbidities, are particularly susceptible to experiencing altered drug metabolism, known as phenoconversion. Phenoconversion is the phenomenon in which an individual's genetically determined metabolism phenotype shifts due to non-genetic factors, such as a normal metabolizer becoming a slow metabolizer. For example, children with sepsis have been reported to exhibit decreased CYP-mediated drug metabolism, antifungals may inhibit CYP metabolism, and certain antimicrobials and antiepileptics can induce metabolism. Research has identified specific drugs that serve as biomarkers for enzymatic rate function within individual CYP pathways. Notable examples include midazolam for CYP3A4, celecoxib for CYP2C9, and omeprazole for CYP2C19. Thus, utilizing the routine administration of preoperative midazolam may serve as a biomarker for assessing CYP3A4 function.

**Methods:**

In our ongoing clinical pharmacogenetic studies, we collected serial serum samples from pediatric burn and surgery patients and utilized liquid chromatography mass spectrometry to measure midazolam and its primary metabolite, OH-midazolam. Preliminary analysis included 80 patient datasets (60 intravenous and 20 oral) to characterize midazolam metabolism by calculating the metabolite-to-parent ratio to identify CYP3A4 pathway induction.

**Results:**

In our initial analysis of 80 patients, eight were identified as outliers, indicating increased metabolism. The median (min-max) ratio was 0.49 (0.01-3.84) for intravenous and 1.34 (0.2-10.50) for oral administration. The patient with the most extreme abnormal midazolam metabolism had severe epilepsy and was taking three different antiseizure medications, likely contributing to induced CYP3A4.

**Conclusions:**

These findings underscore the importance of tailoring drug doses for patients with altered CYP3A4 function. Until further research explores the effectiveness of midazolam as a biomarker for CYP3A4 metabolism, the pursuit of precision medicine will not fulfill its promise in ensuring that all patients receive medication optimized for their dynamic metabolic rates.

**Applicability of Research to Practice:**

This study highlights the potential of midazolam as a clinically valuable biomarker for identifying CYP3A4 phenoconversion.

